# The Effect of Prophylactic Polishing Pastes on Surface Roughness of Indirect Restorative Materials

**DOI:** 10.1155/2014/962764

**Published:** 2014-03-03

**Authors:** Esra Can Say, Haktan Yurdagüven, Özlem Malkondu, Nimet Ünlü, Mübin Soyman, Ender Kazazoğlu

**Affiliations:** ^1^Department of Restorative Dentistry, Faculty of Dentistry, Yeditepe University, Bağdat Caddesi, No. 238, 34728 Göztepe, Istanbul, Turkey; ^2^Department of Prosthodontics, Faculty of Dentistry, Yeditepe University, Istanbul, Turkey; ^3^Department of Restorative Dentistry, Faculty of Dentistry, Necmettin Erbakan University, Konya, Turkey

## Abstract

The purpose of this study was to evaluate the influence of prophylactic polishing pastes (PPP; Detartrine (DT), Topex (TP)) on surface roughness (*R*_*a*_) of indirect composites (IRC; Tescera (TES), Gradia (GRD), and Estenia C&B (EST)), a glass ceramic (Empress 2 layering (E2)), and a leucite reinforced glass ceramic (Empress Esthetic (EE)) with two different (glazed (G); polished (P)) surface preparations. A total of 90 IRC and 120 ceramic discs, 8 mm in diameter and 2 mm thick, were prepared. E2 and EE specimens were randomly divided into two groups (*n* = 30). One group was glazed (GE2; GEE), while the other group was polished (PE2; PEE) the same as the IRCs. The specimens in each group were subsequently divided into three subgroups: control (C), DT, and TP. *R*_*a*_ (*μ*m) was evaluated with a profilometer. Data were analyzed by Kruskal Wallis, followed by the Dunn's multiple comparison tests (*P* < 0.05). DT and TP resulted in significant surface roughening for TES, GRD, and EST, while no significant differences were detected between DT and TP (*P* > 0.05). PE2 and PEE were not affected by DT or TP (*P* > 0.05), while GE2 and GEE exhibited significant roughening after TP (*P* < 0.05). Surface roughness of IRCs and glazed ceramics can be affected by PPP applications.

## 1. Introduction

The surface quality of restorations is one of the most important factors that determines their clinical success in the oral cavity. Surface roughness, gloss, aesthetic appearance, wear resistance, and mechanical properties of the restorations are highly associated with the restorations' surface quality [1, 2]. Among these properties, surface roughness is greatly taken into consideration as roughness has a major impact on the biofilm adhesion and maturation, recurrent caries, gingival irritation [3–7], and staining [8]. Moreover smooth surfaced restorations ensure patient comfort [9] and facilitate oral hygiene [4, 5, 7].

In the oral cavity, restorations are constantly subjected to a variety of factors that may alter their surface roughness [10]. Apart from physiological factors and patient habits [11], individual and professional oral hygiene procedures also play a significant role [10–12]. Studies evaluating the effect of tooth brushing on the deterioration of direct and laboratory-processed indirect composites (IRC) indicated a rapid increase in the surface roughness [10, 12, 13] and decrease in the gloss parameters even though the ceramic materials were considered to be rather inert [10]. Conversely, the effect of professional hygiene maintenance therapies on restorations' surface roughness was expected to be more pronounced, as the prophylactic polishing pastes (PPPs) used during these procedures require a certain amount of abrasiveness for the removal of plaque and stain [12]. The structure of the restorative material, as well as the composition and application procedure of the PPPs, may influence the outcomes [12].

The composition of IRCs is similar to that of direct composites. They are usually classified according to the size of their inorganic fillers. The hybrid IRCs contain fillers that are greater than 1.0 *μ*m, while microhybrid IRCs have fillers smaller than 1.0 *μ*m, and nanohybrid IRCs have fillers smaller than 0.4 *μ*m [14]. IRCs differ from direct composites by the use of various methods of additional polymerization that lead to higher monomer conversion [14]. The additional polymerization procedures can involve photoactivation, heat, pressure, a combination of these methods, or a nitrogen atmosphere [15]. Compared to direct composites, some of these IRCs have improvements in terms of their fatigue behavior [16], mechanical properties [17], and wear resistance [18].

Although comparative studies examining the effect of PPPs on the surface roughness of direct composites and glass ionomers do exist in the literature [12, 19–21], only a few studies have focussed on the influence on indirect restorative materials [21, 22]. Covey et al. [21] reported that the surface roughness of glazed glass ceramic was not affected by the application of PPPs while Yurdaguven et al. indicated significant increase in surface roughness of polished glass ceramic [22]. Therefore, the purpose of this study was to evaluate the influence of two different types of PPPs on the surface roughness of two microhybrid and one hybrid IRCs and to compare them with two reference ceramic materials that were prepared with two surface treatments (glazed versus polished). The hypothesis tested was that PPPs did not affect the surface roughness of the indirect restorative materials.

## 2. Materials and Methods

Three commercially available IRCs, which were chosen in accordance with their different types of filler particles; two microhybrids, Gradia (GRD; GC Europe, Leuven, Belgium) and Tescera (TES; Bisco Inc., Schaumburg IL USA), and one hybrid, Estenia C&B (EST; Kuraray Medical Co, Tokyo, Japan); two ceramic materials: a glass ceramic, Empress 2 layering (E2; Ivoclar Vivadent, Schaan, Liechtenstein), and a leucite reinforced glass ceramic, Empress Esthetic (EE; Ivoclar Vivadent, Schaan, Liechtenstein); and two PPPs: Detartrine (DT; Septodont GmbH, Niederkassel Germany) and Topex (TP; Sultan Healthcare, Hackensack NJ, USA), were used in the study. The properties of the IRCs and ceramics and the composition of the PPPs are listed in Table 1.

A total of 90 IRC discs, 30 from each material (Shade A2), that were 8 mm in diameter and 2 mm thick were prepared. Each material was inserted into a cylindrical metal mold and pressed between two opposing Mylar matrices which were subsequently covered with a 1 mm thick glass slide to extrude the excess material and produce a smooth, flat surface. The specimens were then polymerized through the glass slide using a halogen curing unit (Optilux 501, Kerr Co., Orange, CA, USA) during the initial curing phase. The specimens were further postcured with their proprietary curing units according to their manufacturer's instructions. The TES specimens were placed in a light cycle unit for two minutes (0.5 MPa pressure-light; Tescera ATL Light Cup, Bisco Inc., Schaumburg, IL, USA), followed by a 16-minute heat cycle in water with oxygen scavenger capsules (135°C—Heat Cup, Bisco Inc., Schaumburg, IL, USA) under pressure, light, and heat. GRD specimens were postcured for 5 minutes with a polymerization unit (GC Labolight LV-III, GC Europe, Leuven Belgium). EST specimens were heat-cured for 15 minutes at 110°C (KL 100, Kuraray Medical Corp., Tokyo, Japan). After storage in distilled water for 24 hours at 37°C, the IRC specimens were wet-polished with a sequence of SIC papers (1000-2000-2500 and 3000 grit) followed by the application of 1 *μ*m diamond paste (Diamond Polish, Ultradent, South Jordan, UT, USA) using a polishing machine (Buehler, Lake Bluff, IL, USA) at a rotation speed of 400 rpm.

The E2 (*n* = 60; A2) and EE ceramic specimens (*n* = 60; A2) were prepared with the same diameter and thickness as the IRC specimens according to the manufacturer's instructions. The specimens were then randomly divided into two groups (*n* = 30). One group was glazed (GE2; GEE) with a glazing paste (IPS Empress Universal Glaze Paste; Ivoclar Vivadent, Schaan, Liechtenstein), whereas the other group was polished (PE2; PEE) in the same manner as the IRCs.

The specimens in each group were randomly divided into three subgroups (*n* = 10): (1) Control (C), (2) Detartrine (DT) and (3) Topex (TP) groups. The C group received no treatment. In the DT and TP groups, the specimens were polished with PPP for 12 seconds by renewing the material after 6 seconds. New rotary brushes were used for each specimen with the same low speed hand piece (Kavo 80E, Kavo Dental, Charlotte, NC, USA) at 2000 rpm [20]. The surface roughness of the specimens was evaluated with a profilometer (Perthometer M1 Mahr, Göttingen, Germany). For each specimen, five measurements at different locations and in different directions, with a cut-off length of 0.25 mm, a tracing length of 0.8 mm, and a stylus speed of 0.1 mm/second, were recorded. The roughness value (*R*_*a*_; *μ*m) was calculated as the average of these five readings. During the experimental period, the surface-roughness tester was periodically calibrated (Mahr GmbH, Göttingen, Germany).

### 2.1. Statistical Analysis

The statistical analyses between the control groups of the indirect materials and between the control group and the prophylactic polishing paste applied groups for each material were performed using Kruskal Wallis test, followed by Dunn's multiple comparison test, at a significance level of *P* < 0.05.

### 2.2. Scanning Electron Microscopy

To illustrate the surface characterization of indirect materials and to determine the effects of PPPs, representative scanning electron micrographs (SEM) were taken from each group. The representative SEM specimens had *R*_*a*_ values that were similar to the mean *R*_*a*_ values of their corresponding groups. The specimens were dried, gold-sputter-coated, and observed using a scanning electron microscope (JSM 6335F; JEOL Ltd., Tokyo, Japan).

## 3. Results 

The mean surface roughness (*R*_*a*_; *μ*m), standard deviations, and statistical analysis between the indirect materials and PPP applied groups are shown in Table 2. The comparison between the control groups of the materials revealed that PEE-C showed significantly the lowest *R*_*a*_ values while GE2-C exhibited the highest of all the other materials (*P* < 0.05). No significant differences were found between TES-C, EST-C, or PE2-C (*P* > 0.05) which exhibited significantly lower *R*_*a*_ values than GRD-C and GEE-C (*P* < 0.05).

The application of DT and TP resulted in significant increase in surface roughness for TES (*P* = 0.0001 and *P* = 0.0001, resp.), GRD (*P* = 0.0001 and *P* = 0.0001, resp.), and EST (*P* = 0.0001 and *P* = 0.0001, resp.), while no significant differences in surface roughness were found between TES-DT and TES-TP (*P* = 0.524), GRD-DT and GRD-TP (*P* = 0.607), and EST-DT and EST-TP groups (*P* = 0.585). Conversely, GE2 and GEE showed significant roughening only after TP (*P* = 0.037 and *P* = 0.001) while PE2 and PEE were not affected by the polishing paste applications (*P* = 0.235 and *P* = 0.189, resp.).

### 3.1. Scanning Electron Microscope Observations

**Figure 1:**
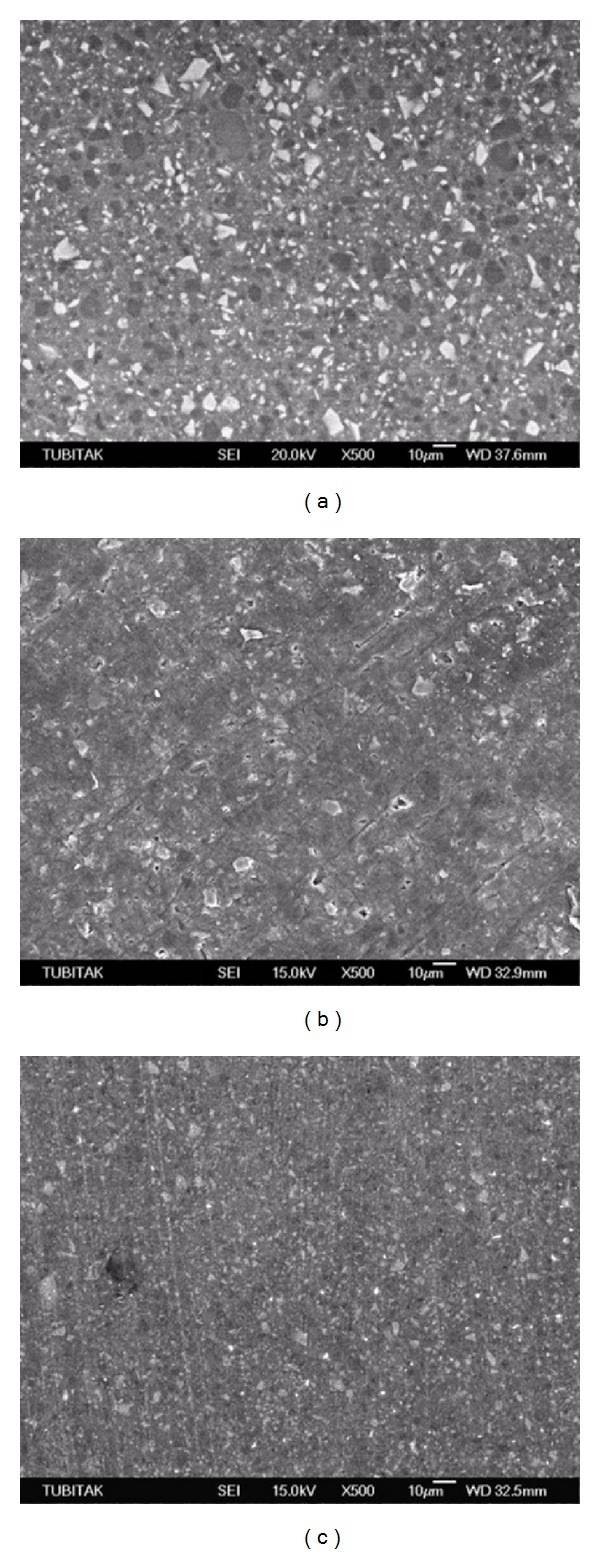
(a) Scanning electron micrograph of TES-C showing a homogenous distribution of different kind of filler particles. (b) TES-DT group presented scratch lines and some debonding of filler particles while TES-TP group revealed smooth scratch lines without debonding of fillers particles (c).

**Figure 2:**
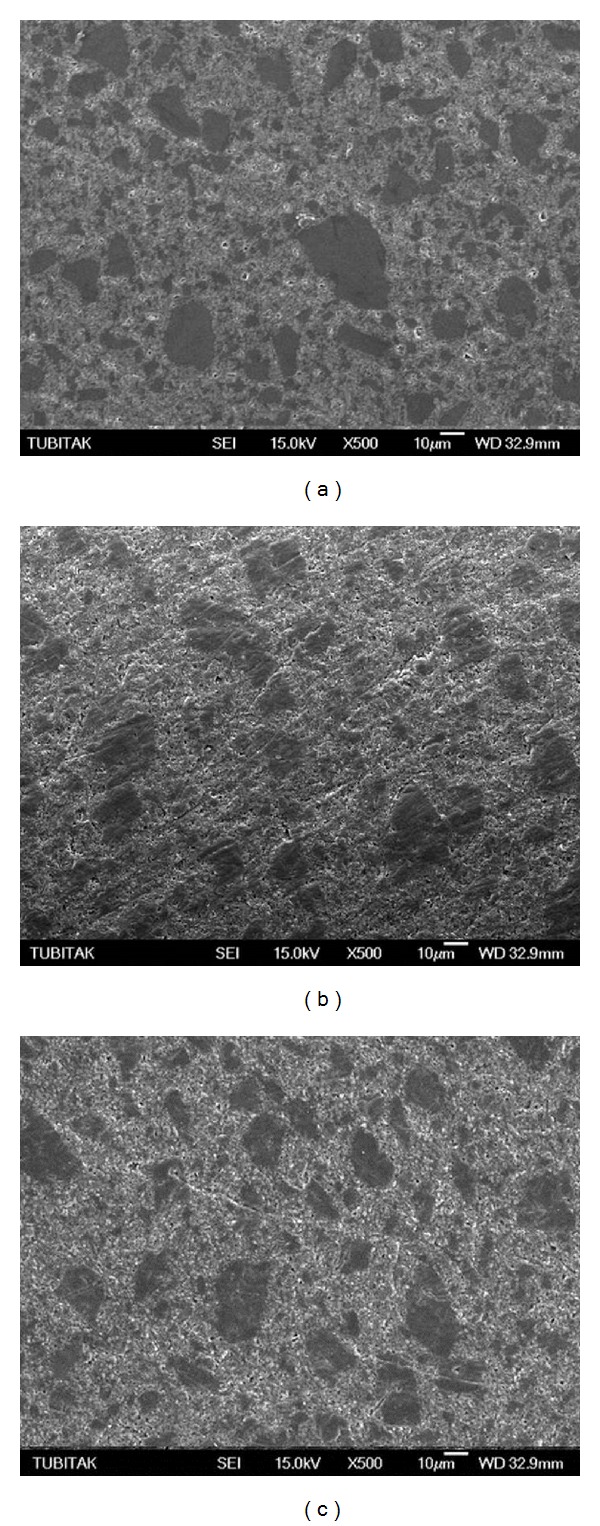
(a) Scanning electron micrograph of GRD-C showed prepolymerized, ceramic, and silica fillers with some debonding. (b) GRD-DT group revealed roughening of prepolymerized fillers along with resin abrasion between the fillers. Debonding of inorganic fillers was also evident. (c) GRD-TP showed similar surface morphology as the GRD-DT, however roughening of prepolymerized fillers was not as much as that in DT group.

**Figure 3:**
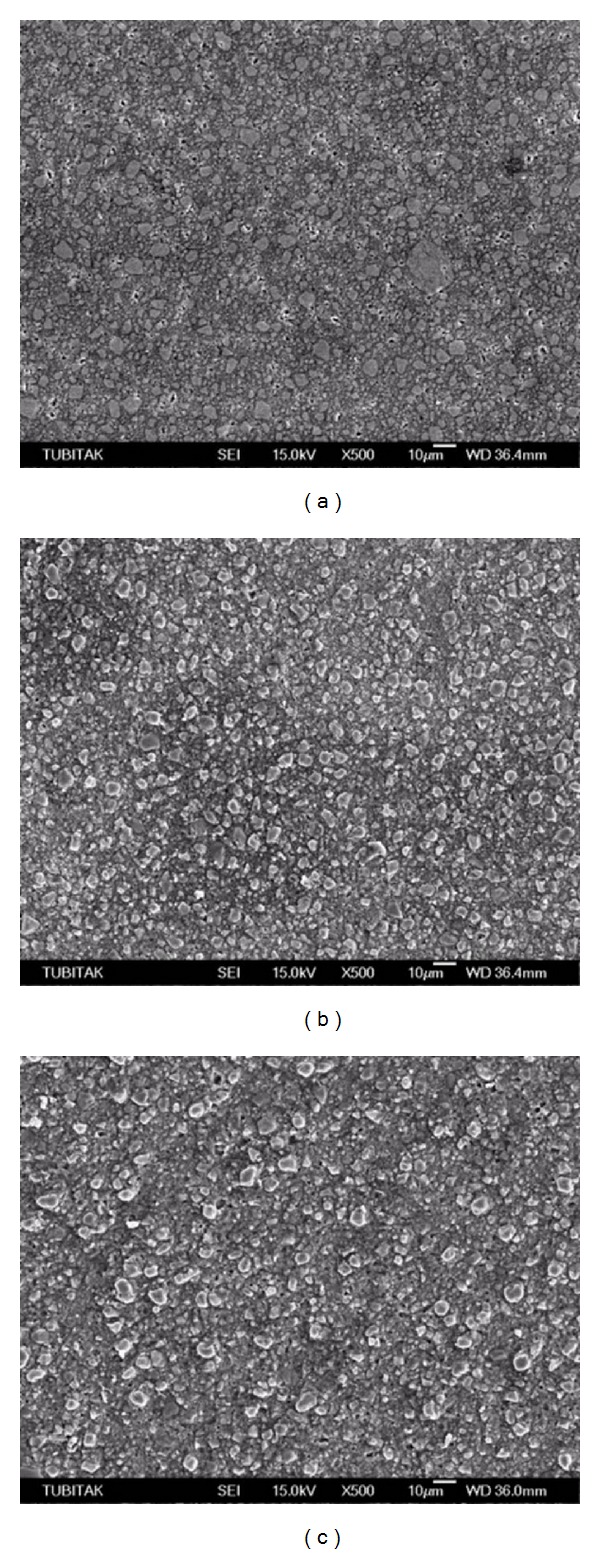
(a) Scanning electron micrograph of EST-C group showed a homogeneous surface with a dense filler distribution and debonding of some inorganic fillers. (b, c) EST-DT and EST-TP groups revealed resin removal between the fillers and debonding of small filler particles.

Scanning electron micrographs of the TES-C group showed a smooth surface with a wide range in particle size variation (Figure 1(a)), while the TES-DT group revealed a few dislodged filler particles and scratch lines (Figure 1(b)). Conversely, the TES-TP group presented smooth scratch lines (Figure 1(c)). GRD-C (Figure 2(a)) revealed prepolymerized and silica fillers with the debonding of some fillers while roughening of prepolymerized fillers and resin abrasion were the characteristic features of the GRD-DT and GRD-TP groups (Figures 2(b) and 2(c), resp.). High density of the inorganic fillers, along with the loss of several fillers, was evident in EST-C (Figure 3(a)). Resin removal and filler protrusion were observed in the EST-DT and EST-TP groups (Figures 3(b) and 3(c), resp.).

**Figure 4:**
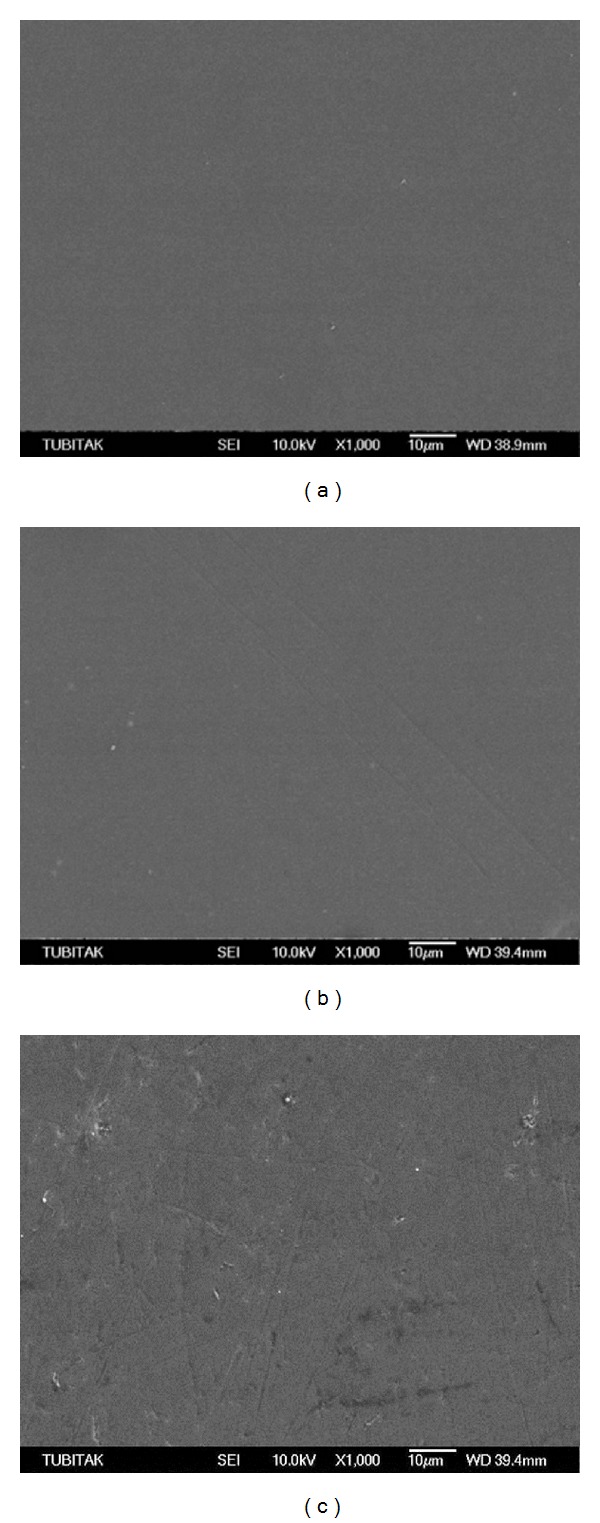
(a) Scanning electron micrograph of GE2-C group showed a homogeneous surface. (b) GE2-DT showed some slight scratch lines while (c) GE2-TP revealed some defects and scratches.

**Figure 5:**
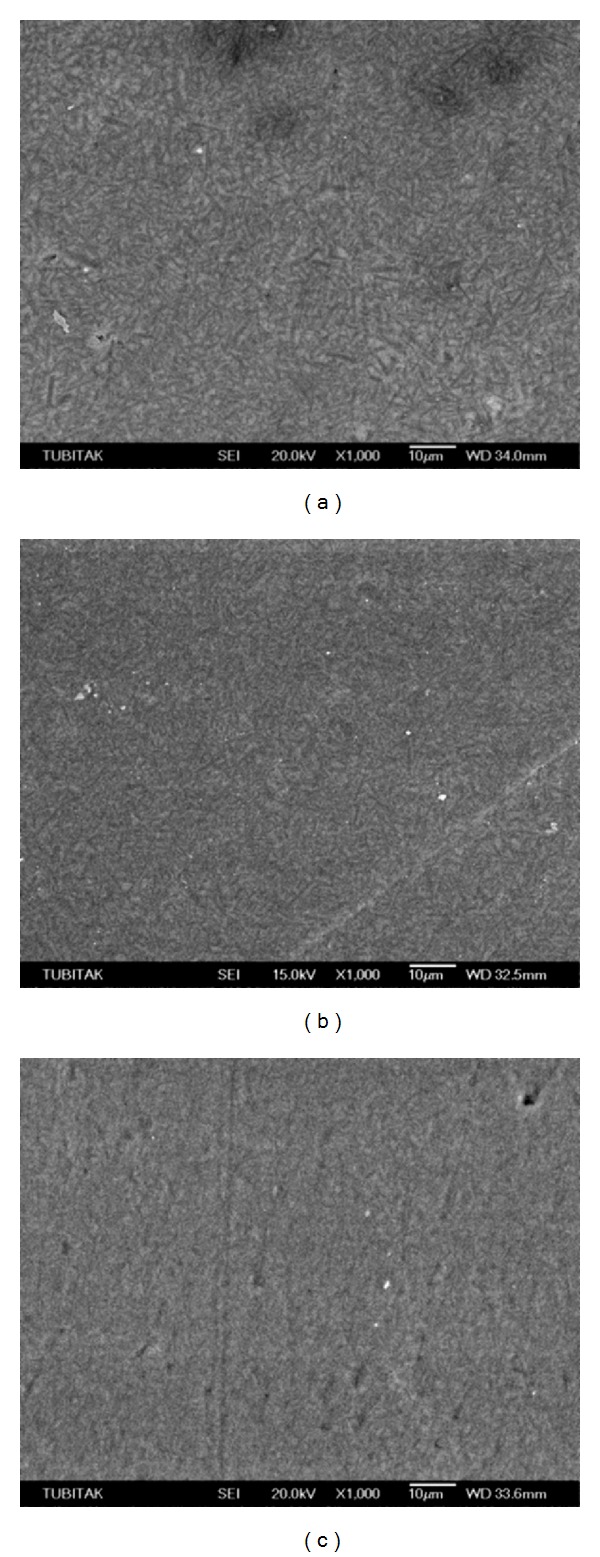
(a, b) Scanning electron micrographs of PE2-C and PE2-DT groups revealed crystals that are characterized by a needle-like morphology while PE2-TP (c) showed some scratch lines.

**Figure 6:**
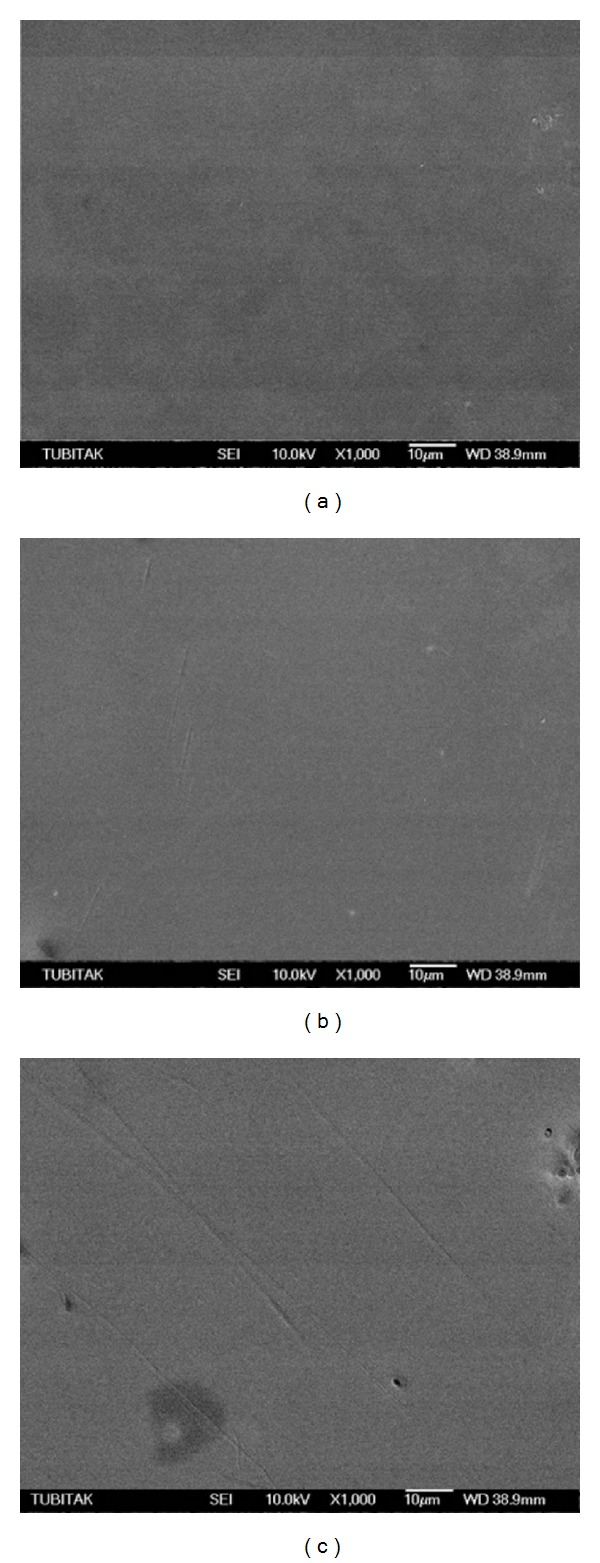
(a, b) Scanning electron micrographs of GEE-C and GEE-DT groups revealed similar homogeneous surfaces while some defects were evident in GEE-TP group (c).

**Figure 7:**
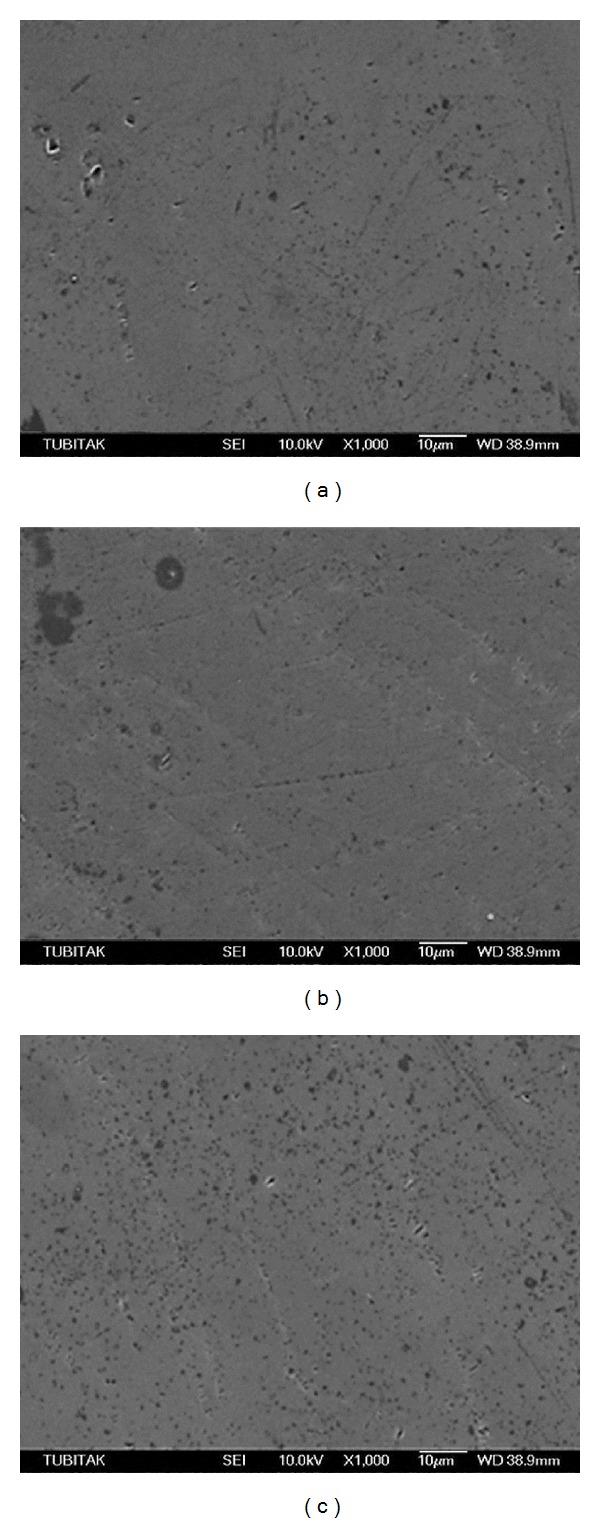
Scanning electron micrographs of PEE-C, PEE-DT, and PEE-TP groups presented similar surface morphology with small voids that were created during mechanical polishing.

GE2-C revealed a homogeneous surface, while GE2-DT showed a number of slight scratch lines (Figures 4(a) and 4(b), resp.). Conversely, GE2-TP group revealed a number of defects and deep scratches (Figure 4(c)). SEM observations of the PE2-C and PE2-DT groups showed crystals that were characterized by a needle-like morphology (Figures 5(a) and 5(b), resp.). In the PE2-TP group, no crystals could be detected; however, some scratches were evident (Figure 5(c)). SEM observation of the GEE-C as well as GEE-DT groups revealed homogeneous surfaces, despite the presence of slight defects in the GEE-TP group (Figures 6(a), 6(b), and 6(c), resp.). The PEE-C, PEE-DT, and PEE-TP groups presented similar surface morphologies with small voids which were created during mechanical polishing (Figures 7(a), 7(b), and 7(c), resp.).

## 4. Discussion 

The cleaning and polishing of teeth and their associated restorations are a part of professional hygiene maintenance therapies. During this procedure, stains and plaque are usually removed by various PPPs that are applied by rubber cups or brushes [23]. Similar to toothpastes, PPPs are typically composed of a binder, humectant, coloring agent, preservatives, fluoride, flavoring, and abrasive grades ranging from coarse to fine [12]. These pastes' mode of action relies upon the physical removal of plaque and stains and they are expected to cause minimal abrasion to dental hard tissues [23] and restorations [12, 20]. However, composites and glass ionomers have been shown to be affected by hygiene maintenance therapies, which increase their surface roughness [12, 20]. Therefore, this study aimed to investigate the effects of two commercially available PPPs on the surface roughness of three IRCs and to compare them with two reference ceramic materials that were prepared with two surface treatments (glazed versus polished).

Prior to the application of PPPs, all IRCs and ceramic specimens were polished up to 3000 grit SIC paper which has the same grit size (5 *μ*m) [24] as the finest discs of the Sof lex polishing system [25]. This method was employed to represent clinically relevant polishing regimens. PPP applications on the specimens were carried out by a single operator, using a slow speed hand piece at 2,000 rpm, and the treatment time was fixed at 12 seconds, as recommended by Yap et al. [20]. To eliminate interindividual differences, a second operator, who was blind to both the materials and the polishing pastes, performed all roughness evaluations.

The results of the study indicate that the null hypothesis, that is, PPP applications do not affect the surface roughness of indirect materials, was only accepted for the polished ceramics PE2 and PEE. The application of PPPs significantly increased the surface roughness of the IRCs, TES, GRD, and EST (*P* < 0.05), while the difference between the two PPPs was not significant (*P* > 0.05). Conversely, the glazed ceramics GE2 and GEE showed significant roughening after TP (*P* < 0.05; Table 2).

PPPs serve as a third body between the restoration and the polishing instrument, abrading composite surfaces with a three-body wear process [12]. Three-body wear involves the loss of resin matrix between filler particles and the subsequent dislodgement or debonding of filler particles, which may result in an even rougher surface [26]. The resin matrix is selectively removed, especially when the abrasives of the PPPs are harder than the resin matrix of the composites [20]. The PPP, DT, contained silica as the abrasive and a 35% formaldehyde solution, whereas the ingredients of TP were 1.23% APF and an 8 to 10 *μ*m particle sized abrasive. Although all of the IRCs, TES, GRD, and EST showed significant roughening after the applications of both PPPs and no significant differences were observed between their *R*_*a*_ values, they differed from each other in their surface responses. The hybrid-ceramic IRC, EST, has a four-functional urethane methacrylate (UTMA) resin matrix. Resin removal and some filler debonding were observed on SEM after the application of DT and TP (Figures 3(b) and 3(c)). The pronounced exposure of filler particles contributed to the increase in *R*_*a*_ values of this IRC (Table 2). In fact, its higher filler content (92% wt) is expected to protect the resin matrix from excessive abrasion, resulting in smoother surfaces [27]; however, fillers that are much harder than the resin matrix may cause prominent matrix abrasion during polishing [28]. Conversely, the microhybrid IRC, TES, consists of an EBis-GMA and a two-functional urethane methacrylate (UDMA) resin matrix, and in contrast to EST the application of PPPs resulted in deep, smooth scratch lines (Figures 1(b) and 1(c), resp.), as well as debonding of filler particles (Figure 1(b)). The SEM images of this IRC showed neither resin removal nor filler protrusions. Conversely, the microhybrid IRC, GRD, contains a two-functional urethane methacrylate (UDMA) and an EDMA-based organic matrix. SEM analysis revealed the roughening of the prepolymerized fillers that had a lower hardness than the glass fillers [29], as well as some debonding and resin abrasion between the fillers. The differences in the surface responses of the indirect composites observed on SEM can be attributed to the types of inorganic fillers and the type and ultimate degree of cure of the resin matrix [30].

During glazing, ceramic powder is applied on the ceramic surfaces and heated up to a temperature close to that of firing [31]. However, occlusal checking and adjustment of adhesively luted ceramic restorations can only be performed after the cementation procedure, which mostly removes the ideal glazing. In these circumstances, ceramic surfaces are mechanically polished by various polishing instruments and diamond pastes. In this study, to represent the clinical conditions, PPPs were applied on both the glazed and polished surfaces of E2 and EE. E2 is a sintered fluoroapatite glass ceramic that is used for veneering the heat-pressed, lithium disilicate glass ceramic Empress 2 [32]. On the other hand, EE is a leucite reinforced glass ceramic [33]. EE revealed significantly lower *R*_*a*_ values than E2 with both of the surface treatments (glazed versus polished). Similarly, Olivera and Marques [34] observed lower *R*_*a*_ values for the leucite reinforced ceramic IPS Empress than the layering glass ceramic Empress 2. Conversely, GE2-C and GEE-C showed significantly higher *R*_*a*_ values than their polished counterparts (Figures 4(a), 6(a), 5(a), and 7(a), resp.); this result is consistent with the findings in the literature [13]. This finding could be attributed to glazing, which could produce an undulating, rough, and irregular surface [35].

TP contains 1.23% acidulated phosphate fluoride (APF). The ingredients of APF are sodium fluoride, phosphoric acid, and H^+^ and F^−^ ions. The hydrofluoric acid in APF dissolves the silica in the ceramic, which results in a loss of mass and increased surface roughness [36]. The degree of surface damage to the ceramics varies with the type of ceramic or glaze used [37]. The low ceramic content of the glazed layer (60–70% ceramic powder and pigments, 30–40% glycol) may render the glazed ceramics, GE2 and GEE, more susceptible to etching and scratching by APF in TP, compared to the polished ceramics, PE2 and PEE, which correlated well with the SEM observations (Figures 4(c), 6(c), 5(c), and 7(c), resp.). This result is also consistent with the studies in the literature that showed that polishing of dental ceramics reduced the effects of APF [35–37].

One of the main failures of indirect restorations is the formation of secondary caries [38], which result from plaque accumulation that is subsequently aggravated by the surface roughness of the restorative material [39]. Based on studies using mechanical profilometry devices, the critical threshold value of surface roughness for the simultaneous increase in plaque accumulation is 0.2 *μ*m. Any further increase above this critical value increases the risk for caries and periodontal inflammation [4]. In this study, PPPs applications were administered for a short time to minimize their effects and the subsequent volume loss. However, even this approach increased the surface roughness of the IRCs, TES, GRD, and EST, above the critical threshold value. Under the dynamic conditions of the oral environment and with subsequent use of the applications the increase in surface roughness is expected to be more pronounced [20]. Therefore, clinicians should decide whether to avoid the polishing of IRCs during hygiene maintenance therapies, apply a protective barrier, or further repolish the IRCs with low grit polishing pastes. Conversely, regarding GE2 and GEE, TP application resulted in a significant increase in *R*_*a*_ values; however this roughness did not reach the critical threshold value. Nevertheless, similar to composite materials, ceramic materials are also affected by stress and dynamic fatigue in the oral environment. The degradation of the surface may in turn influence the materials' physical and mechanical properties [40]. Therefore, any procedure that may further accelerate this degradation should be considered with caution.

## 5. Conclusions

The following conclusions can be drawn based on the results of this study.

1. The application of prophylactic polishing pastes increased the surface roughness of the indirect composites, TES, GRD, and EST, above the critical threshold value for plaque accumulation.
2. The surface roughness of the polished glass ceramic, PE2, and leucite reinforced glass ceramic, PEE, was not affected by the prophylactic polishing paste applications, which depended on the type of the paste; the surface roughness of the glazed glass ceramic, GE2, and the glazed leucite reinforced glass ceramic, GEE, were significantly influenced.
3. Indirect composite restorations may result in greater aesthetic and biological disadvantages than ceramic restorations.

## Figures and Tables

**Table 1:** The characteristics of the materials used in the study.

Materials	Type	Filler type	Content filler load (% by weight)	Resin system	Curing type	Lot	Manufacturer
Tescera (TES)	Microhybrid IRC	Glass filler, Amorphous silica	81%	EBis-GMA, UDMA	Light/heat/pressure	0600001607	Bisco Inc., Schaumburg, IL, USA

Gradia (GRD)	Microhybrid IRC	Ceramic, pre-polymerized fillers, silica	75%	EDMA, UDMA	Light	1202251	GC Europe, Leuven, Belgium

Estenia C&B (EST)	Hybrid-ceramic IRC	Surface treated alumina microfiller, Silanated glass ceramics	92%	UTMA, Hydrophobic aromatic dimethacrylate Hydrophobic aliphatic dimethacrylate	Light/Heat	00010A	Kuraray Medical Co., Tokyo, Japan

Empress 2 layering (E2)	Glass ceramic	SiO_2_, Al_2_O_3_, P_2_O_5_, K_2_O, Na_2_O, CaO, F, and pigments				G09402	Ivoclar Vivadent, Schaan, Liechtenstein

Empress Esthetic (EE)	Leucite reinforced glass ceramic	SiO_2_, K_2_O, Al_2_O_3_, Na_2_O, CaO, others oxide, and pigments				P68613	Ivoclar Vivadent, Schaan, Liechtenstein

Detartrine (DT)	Prophylactic polishing paste	Silica, 35% formaldehyde solution				B05737AA	Septodont GmbH, Niederkassel, Germany

Topex (TP)	Prophylactic polishing paste	1.23% APF, 8–10 µm particle sized abrasive				041811F	Sultan Healthcare, Hackensack, NJ, USA

EBis-GMA: ethoxylated bis-GMA; UDMA: urethane dimethacrylate; UTMA: urethane tetramethacrylate; Bis-GMA: bisphenol-A-glycidyl dimethacrylate.

**Table 2:** Mean surface roughness (*R*_*a*_; *µ*m), standard deviations (±sd), and statistical analysis of the control and prophylactic polishing paste (PPP) applied indirect restorative materials.

Indirect restorative materials	Control (C)	Detartrine (DT)	Topex (TP)
TES	0.036 ± 0.004^A,a^	0.232 ± 0.019^b^	0.248 ± 0.032^b^
GRD	0.049 ± 0.007^B,c^	0.207 ± 0.035^d^	0.213 ± 0.039^d^
EST	0.036 ± 0.003^A,e^	0.206 ± 0.056^f^	0.212 ± 0.037^f^
GE2	0.115 ± 0.010^D,g^	0.111 ± 0.012^g^	0.181 ± 0.011^h^
PE2	0.033 ± 0.019^A,i^	0.040 ± 0.011^i^	0.050 ± 0.015^i^
GEE	0.061 ± 0.016^C,j^	0.072 ± 0.019^j^	0.113 ± 0.02^k^
PEE	0.017 ± 0.006^E,l^	0.020 ± 0.005^l^	0.019 ± 0.004^l^

Different capital superscript letters in the control column indicated significant differences between the control groups, while different superscript letters in the same row showed significant differences between control and PPPs applied groups for the same material (*P* < 0.05).
